# Characterization of *Erysiphe necator*-Responsive Genes in Chinese Wild *Vitis quinquangularis*

**DOI:** 10.3390/ijms130911497

**Published:** 2012-09-12

**Authors:** Min Gao, Jiao Niu, Suping Zhao, Chen Jiao, Weirong Xu, Zhangjun Fei, Xiping Wang

**Affiliations:** 1 College of Horticulture, Key Laboratory of Horticultural Plant Biology and Germplasm Innovation in Northwest China, Ministry of Agriculture, Northwest A & F University, Yangling, Shaanxi 712100, China; E-Mails: gaomin.001@163.com (M.G.); niujiao.2009@163.com (J.N.); bashijiu@163.com (S.Z.); dd_race@sohu.com (C.J.); 2 State Key Laboratory of Crop Stress Biology in Arid Areas, Northwest A & F University, Yangling, Shaanxi 712100, China; 3 School of Agriculture, Ningxia University, Yinchuan, Ningxia 750021, China; E-Mail: xwr9320@163.com; 4 Boyce Thompson Institute, Cornell University, Ithaca, NY 14853, USA; E-Mail: zf25@cornell.edu; 5 USDA Robert W. Holley Center for Agriculture and Health, Ithaca, NY 14853, USA

**Keywords:** Chinese wild *Vitis quinquangularis*, *Erysiphe necator*, SSH, EST, qRT-PCR

## Abstract

Powdery mildew (PM), caused by fungus *Erysiphe necator*, is one of the most devastating diseases of grapevine. To better understand grapevine-PM interaction and provide candidate resources for grapevine breeding, a suppression subtractive hybridization (SSH) cDNA library was constructed from *E. necator*-infected leaves of a resistant Chinese wild *Vitis quinquangularis* clone “Shang-24”. A total of 492 high quality expressed sequence tags (ESTs) were obtained and assembled into 266 unigenes. Gene ontology (GO) analysis indicated that 188 unigenes could be assigned with at least one GO term in the biological process category, and 176 in the molecular function category. Sequence analysis showed that a large number of these genes were homologous to those involved in defense responses. Genes involved in metabolism, photosynthesis, transport and signal transduction were also enriched in the library. Expression analysis of 13 selected genes by qRT-PCR revealed that most were induced more quickly and intensely in the resistant material “Shang-24” than in the sensitive *V. pseudoreticulata* clone “Hunan-1” by *E. necator* infection. The ESTs reported here provide new clues to understand the disease-resistance mechanism in Chinese wild grapevine species and may enable us to investigate *E. necator*-responsive genes involved in PM resistance in grapevine germplasm.

## 1. Introduction

Grapevine (*Vitis vinifera* L.) is the most widely-grown fruit crop worldwide and used for wine, table grape, and dry fruit production. Powdery mildew (PM) caused by *Erysiphe necator* (Schw.) Burr., is one of the major fungal diseases in grapes, resulting in huge losses of berry quality and grape production. Due to the high cost and toxicity associated with fungicide application, developing resistant cultivars using wild species could be an efficient, economical, and environmentally friendly strategy to reduce the threat of the disease. Therefore, understanding the mechanisms of PM resistance, identifying and functionally characterizing key genes in the resistant germplasm may provide candidate genes and valuable information on transgenic engineering for improving plant resistance to PM and withstanding harsh conditions.

Several recent studies have been carried out to investigate the biology of the infection process of grapevine by *E. necator* and to characterize defense reactions in *Muscadinia rotundifolia* [1–3], North American grapevine species [4] and European group grapevines [4,5]. Resistance to PM in *M. rotundifolia* could also be traced back to *Run*1, a single dominant locus that contains two families of resistance gene analogs [2,3]. The constitutive expression of pathogenesis-related (PR) genes, such as PR-2 and PR-3 in disease-resistant North American grapevine species [4], may represent one type of QTL-mediated cumulative effect. Fung *et al.* [4] reported that only three transcripts in *V. aestivalis* and 625 in *V. vinifera* were identified to be involved in PM-responsive process. Fekete *et al.* [1] identified 25 disease-response genes in *V. vinifera* Cabernet Sauvignon and found that they were up-regulated specifically in response to *E. necator*. These previous studies provide valuable information on the interaction between PM and European and North American grapevines. However, little is known about the transcriptional and differential gene expression patterns upon PM-inoculation of wild grapevine species originating from China.

China is one of the major centers of origin of *Vitis* species [6]. Among the nearly 70 known species in *Vitis*, more than 27 have their origins from China [6,7]. These wild *Vitis* species have been systematically evaluated for PM resistance by natural and artificial identification in field conditions when disease symptoms were fully developed [6]. Among them, Chinese wild *V. quinquangularis* clone “Shang-24” has been reported to be highly resistant to *E. necator* [7]. Discovery of a highly PM-resistant trait in Chinese wild *Vitis* species provides a valuable resource and germplasm for genetic improvement and molecular analysis of the PM resistance mechanism.

Subtractive suppression hybridization (SSH) is an effective method that can be used to maximize the identification of genes involved in host responses to pathogen infection and disease development. This technique has been successfully used to isolate functional genes that are responsive to pathogen infection in plants [8–10]. In this study, to better understand the molecular basis of the high resistance to PM by Chinese wild *V. quinquangularis*, we constructed an SSH cDNA library to determine the differential expression of PM-responsive genes.

## 2. Results

### 2.1. *E. necator* Developmental Stages

**Figure 1:**
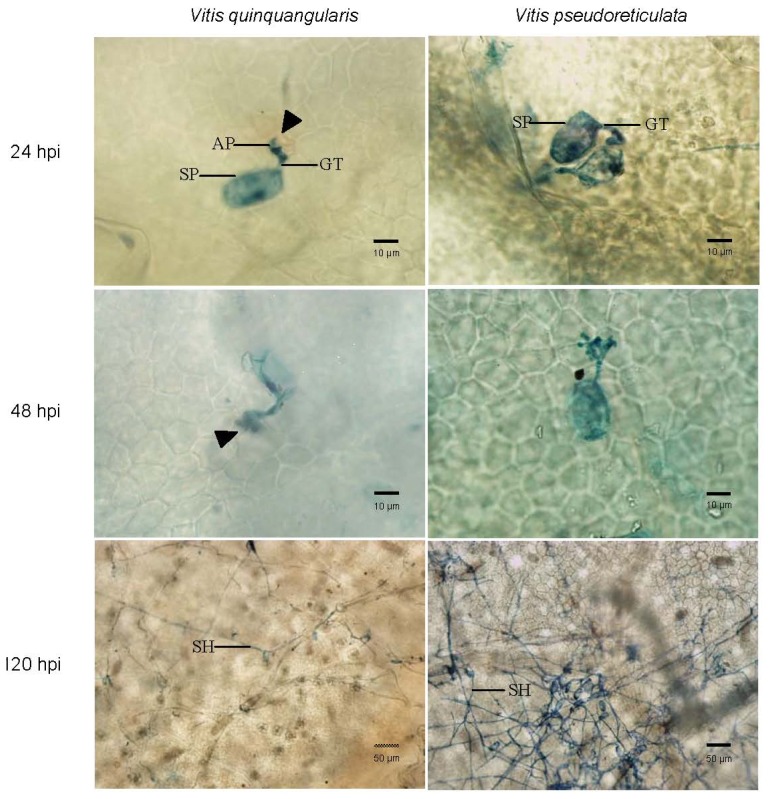
Progression of PM on *V. quinquangularis* clone “Shang-24” and *V. pseudoreticulata* clone “Hunan-1” leaves. Shown are representative images taken at 24, 48, and 120 hpi with conidiospores. Spores and hyphae were stained with aniline blue (0.1% *v*/*v* in lacto glycerol). H_2_O_2_ accumulation was indicated by brown staining due to DAB polymerization. Micrographs were obtained under transmitted white light. Bars show scale. Filled triangles mark epidermal cell browning beneath the appressorium; SP: conidiospore; GT: germ tube; AP: appressorium; SH: secondary hyphae.

To investigate the developmental time-course of the pathogen in Chinese wild *V. quinquangularis* clone “Shang-24” and *V. pseudoreticulata* clone “Hunan-1”, we conducted a microscopy study of conidiospore germination and hyphal development during a 5-day time period. Microscopic images of 24, 48 and 120 hpi are presented in Figure 1.

Histological observations revealed that conidial germination, germ tube formation, and development of the appressorium occurred on leaves of both grapevine clones (Figure 1). At 24 hpi, conidiospores produced germ tube and appressoria on both *V. quinquangularis* and *V. pseudoreticulata* leaves. In *V. quinquangularis* leaves, most epidermal cells invaded by the conidiospores led to a hypersensitive response and the attachment by the conidiospores induced H_2_O_2_ accumulation, as indicated by brown staining due to DAB polymerization (Figure 1). At this stage, the staining was obvious but faint and it appeared to be in the wall of mesophyll cells where they made contact with the attacked epidermal cells. Mesophyll that had no contact with the attacked epidermal cells did not show any brown staining, and the staining was intense on reaching 120 hpi in *V. quinquangularis* leaves. In contrast, in *V. pseudoreticulata* leaves during the early phase of infection, no DAB staining appeared, and even at 120 hpi only very weak DAB staining was detected in mesophyll cells at few infection sites. The further infection led to the formation of colonies with dense secondary hyphae on *V. pseudoreticulata* leaves, but only small colonies with sparse hyphae on *V. quinquangularis* leaves at 120 hpi (Figure 1). On the basis that the first infection occurred within 24 hpi in Chinese wild *V. quinquangularis* “Shang-24” (Figure 1), in the present study we chose leaves at 12 to 120 hpi for the SSH library construction.

### 2.2. SSH Library Construction, EST Sequencing, Assembly, and Annotation

**Figure 2:**
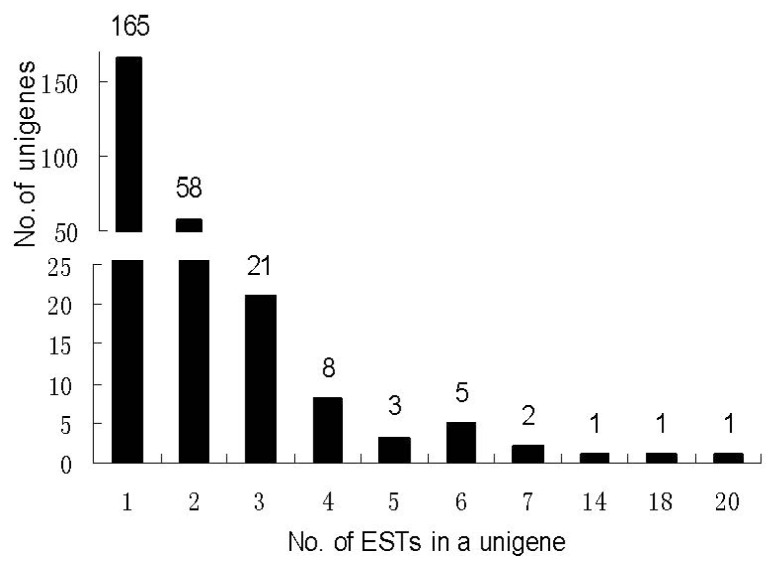
Distribution of number of EST members in each grape unigene.

To identify genes that are potentially involved in *V. quinquangularis* clone “Shang-24” resistance to *E. necator*, an SSH library was constructed with mock-inoculated “Shang-24” leaves as the driver and *E. necator*-inoculated leaves as the tester. A total of 605 clones from the SSH library were selected and PCR analysis of white *E. coli* colonies containing cDNA inserts in the pGEM-T Easy vector showed that the size of the cDNA inserts ranged from 150 to 1000 bp. This confirmed the quality of the subtracted cDNA library. A total of 526 clones were identified to contain inserts larger than 200 bp, and were selected for sequencing. After removing low quality sequences and sequences of bacterial origin, 492 high quality ESTs ranging between 200 and 1000 bp were obtained. These ESTs were further assembled into 266 unigenes, among which 101 were contigs and 165 were singletons (Table S1). The number of EST members in unigenes varied from one to 20 (Figure 2), with the majority of unigenes present in low copy numbers.

The “Shang-24” unigenes were further analyzed by searching against the NCBI (National Center for Biotechnology Information) nr database using the BLASTX program. A total of 205 (77.1%) unigenes including 77 contigs and 128 singletons showed significant similarities to known functional gene sequences in the database, 25 had matches against genes of unknown function or hypothetic proteins. The remaining 36 sequences (15 contigs and 21 singletons) did not match to any known gene sequences in the database.

**Figure 3:**
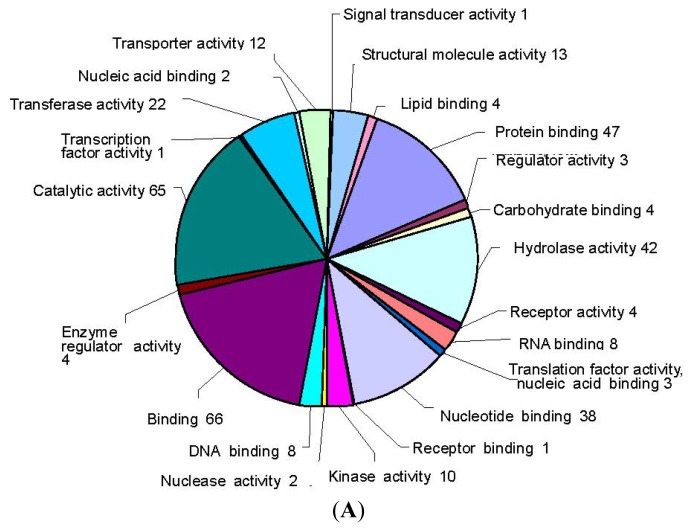

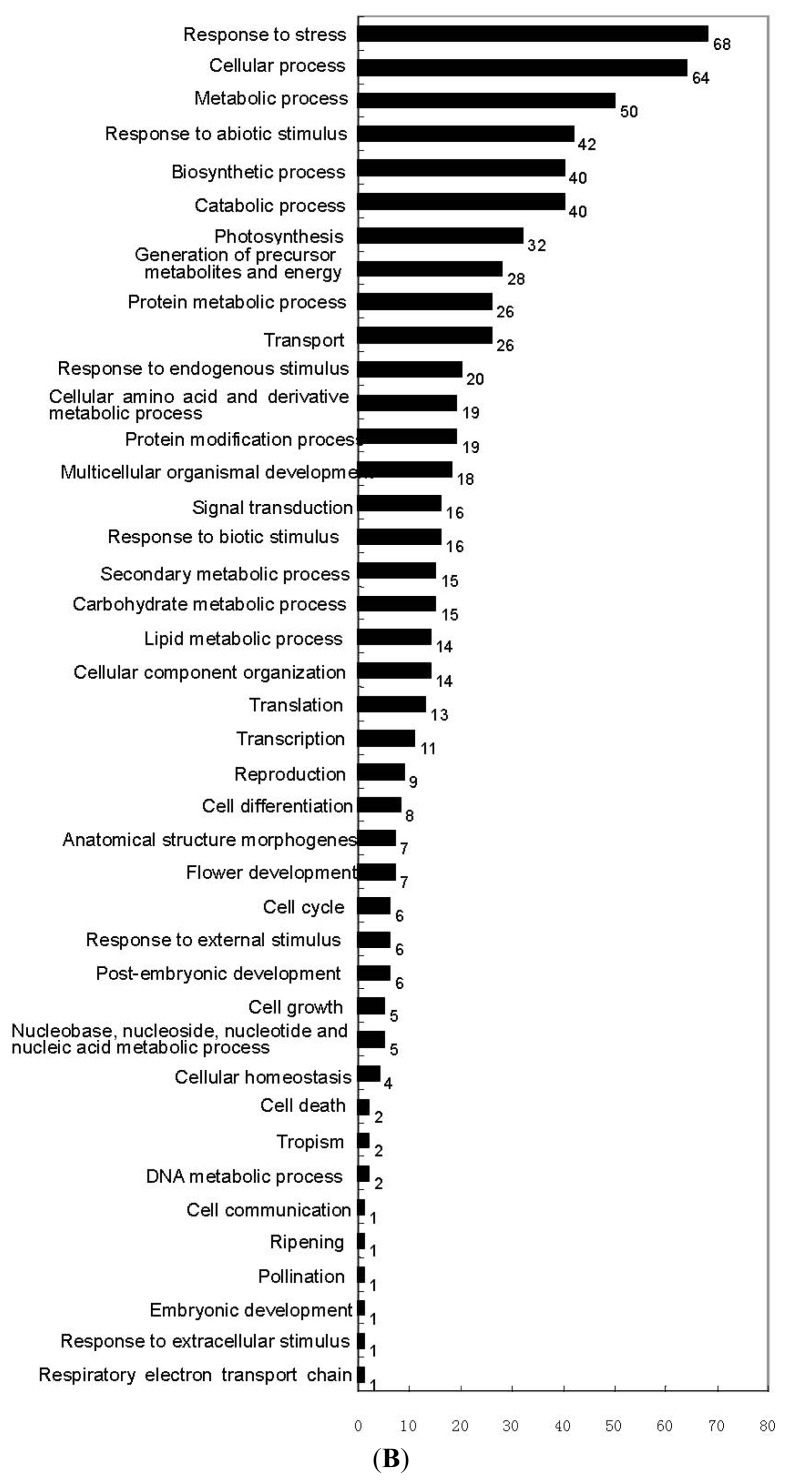
Functional classification of *V. quinquangularis* “Shang-24” unigenes within (**A**) molecular function categories and (**B**) biological process categories, respectively.

The unigenes from *E. necator*-inoculated grapevine leaves were further assigned gene ontology (GO) terms. Based on the GO annotations, unigenes were classified into high-level plant specific GO slims within the two ontology categories, namely, molecular function and biological process. A total of 176 unigenes were assigned to 22 GO slims within the molecular function category and 188 unigenes to 41 GO slims within the biological process category (Figure 3). Within the molecular function category, catalytic activity and binding were the most abundant GO slims (Figure 3A). Response to stress was the most abundant GO slim within the biological process category, which contained responses to stress, abiotic stimulus, and chemical stimulus, which is consistent with the fact that the unigenes were derived from tissues subjected to *E. necator* inoculation in Chinese wild *V. quinquangularis* (Figure 3B).

### 2.3. PM Resistance-Related Genes

Although a large amount of *E. necator*-induced sequence information was generated from Chinese wild *V. quinquangularis* in this study, only 87 unigenes that were thought to be more relevant to PM resistance because of their defense-related roles and the higher number of those genes were sorted into different categories, based on their putative functions (Table 1). The largest category, consisting of 22 (8.3%) unigenes, was predicted to encode resistance and stress-related proteins, such as PR protein, senescence-associated protein, glutathione *S*-transferase 3, F-box family protein, chitinase, proline-rich protein, and catalase. Nineteen (7.1%) unigenes were related to metabolism, 15 (5.6%) encoding proteins and enzymes putatively involved in photosynthesis and energy, the majority of which encoded ribulose-1, 5-bisphosphate carboxylase/oxygenase activase, 7 (2.6%) unigenes were related to protein synthesis and fate, and genes involved in signal transduction, transport, and transcription accounted for 3.0%, 4.5%, and 1.5%, respectively.

### 2.4. Expression Analysis of *E. necator-*Responsive Genes by qRT-PCR

**Figure 4:**
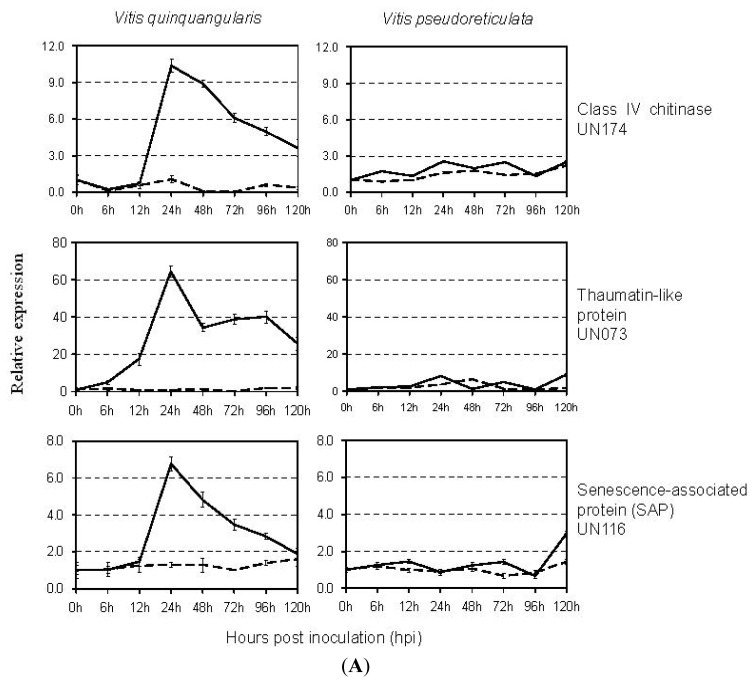

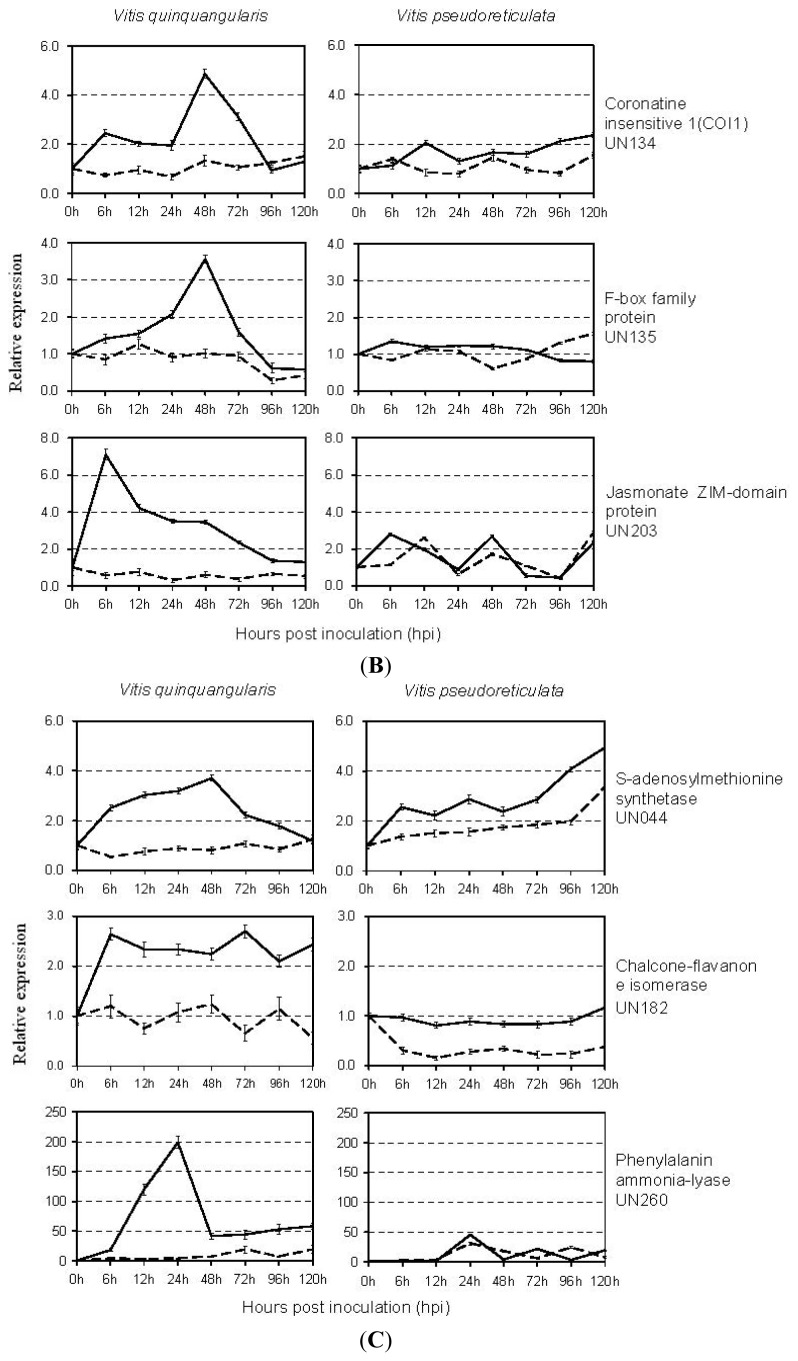

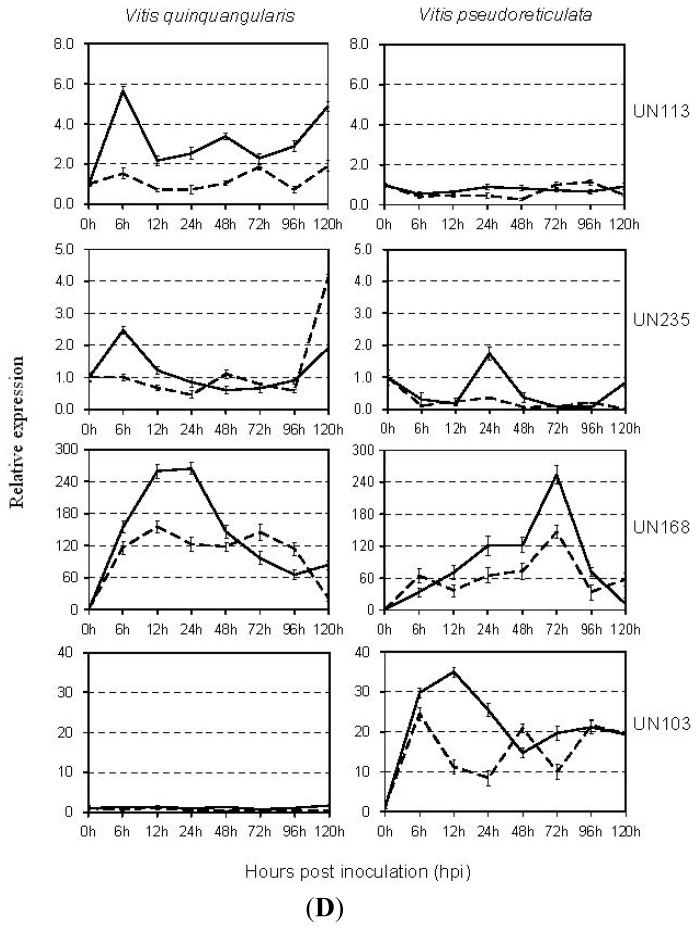
Real-time quantitative RT-PCR analysis of transcript accumulation in two grapevine genotypes in response to *E. necator* at 0, 6, 12, 24, 48, 72, 96, and 120 hpi. Data represents means of triplicate data. Expression profiles of genes in the disease/defense category (**A**); genes involved in Ubiquitin/26S proteasome pathway (**B**); genes in the metabolism category (**C**); and genes with no known homologs (**D**) are shown. Solid line, *E. necator*-inoculated samples; dashed line, mock-inoculated samples.

To further confirm the expression patterns of identified genes during the interaction between grapevine and *E. necator* at different time points, qRT-PCR was performed with 11 genes represented by one EST and two genes by two or more ESTs on *E. necator*- and mock-inoculated samples at eight time points (0, 6, 12, 24, 48, 72, 96, and 120 hpi). These 13 genes in different functional groups were selected for the qRT-PCR analysis because of defense-related roles of their homologs in hypersensitivity, ubiquitin/proteasome pathway and phenylpropanoid synthesis reported previously, and because of their novel sequences. Nine of them corresponded to three functional categories: metabolism (3 genes), protein synthesis and fate (3 genes) and response to defense (3 genes) (Table 1), and four had no match to any sequences in the GenBank nr database. Distinct expression patterns of these genes were observed in resistant and susceptible grapevines in response to *E. necator* and most of the selected genes were strongly elevated by *E. necator* only in the resistant material “Shang-24”, while not in the susceptible material “Hunan-1” (Figure 4).

Three genes involved in defense (UN073, UN116, and UN174), which encode a thaumatin-like protein, a senescence-associated protein, and a class IV chitinase, respectively, shared similar expression patterns in the resistant plant. They did not start increasing until 12 hpi, and all of them reached their maximum expression levels at 24 hpi, then decreased gradually from 48 to 120 hpi; while in the susceptible material, the expression levels of these three genes were consistently close to the control levels throughout the entire time course (Figure 4A).

Three genes in the protein synthesis and fate group (UN134, UN135, and UN203), coding for a coronatine insensitive 1 (COI1), an F-box family protein, and a jasmonate ZIM-domain protein, respectively, were activated strongly by *E. necator* from 6 to 72 hpi in the resistant material “Shang-24”, while in “Hunan-1”, their transcripts consistently remained at control levels throughout the entire time course (Figure 4B).

Three genes in the category of metabolism (UN044, UN182, and UN260) showed homology to *S*-adenosylmethionine synthetase, chalcone-flavanone isomerase, and phenylalanine ammonia-lyase, respectively (Table 1). They all sharply increased as early as at 6 hpi in “Shang-24” and reached the maximal levels at 48 hpi, whereas their expression did not change significantly in “Hunan-1” upon *E. necator*-inoculation (Figure 4C).

The expression pattern of four novel genes that had no hits in the database at different time points post inoculation of *E. necator* is shown in Figure 4D. Two genes (UN113 and UN235) were induced as early as 6 hpi in “Shang-24”, but remained at control levels (UN113) or did not increase until 24 hpi (UN235) in “Hunan-1”. Another gene (UN168) increased at 6 hpi and reached the maximum expression level at 24 hpi in “Shang-24” in response to *E. necator*, while in “Hunan-1”, the reaction was delayed until 72 hpi. Gene UN103 showed a completely different expression pattern with other genes in our experiment: induced at 6 hpi and reached its maximum expression level at 12 hpi in “Hunan-1” though also slightly increased in “Shang-24” (Figure 4D).

## 3. Discussion

### 3.1. Analysis of *E. Necator* Developmental Stages

Powdery mildew fungus enters the epidermal cells of the leaf via conidiospores and the formation of a primary hypha is an indication of successful cell penetration and the establishment of a compatible interaction. Conidiospores produced germ tube and appressoria on both *V. quinquangularis* and *V. pseudoreticulata* leaves at 24 hpi, and a similar finding was reported in a previous study [4]. Recently, a microscopy study of the infection process of *E. necator* showed that appressorium formation, host cell penetration, and primary hypha formation took place during the period from 12 to 48 hpi in the susceptible *V. vinifera* Cabernet Sauvignon [1].

Restriction of *E. necator* growth in *V. quinquangularis* is a post-infection phenomenon that begins when the first haustoria enters mesophyll cells, resulting in the necrosis of guard cells, the accumulation of peroxidases, and in some cases a hypersensitive reaction depending on environmental conditions [11,12]. This correlates well with the specific induction of genes related to hypersensitivity and phenylpropanoid synthesis (Figure 4). Pathogen spread was severely impaired between 48 and 120 hpi in *V. quinquangularis*, suggesting that the resistance mechanism is already in effect before this time point, consistent with the strong transcriptional reprogramming observed before 48 hpi (Figure 4).

### 3.2. Construction of an SSH Library after *E. Necator* Inoculation

As a valuable tool in functional genomics, SSH has been applied to maximize the identification of genes involved in host responses to pathogen infection and disease development. In this study, a PCR-based cDNA subtractive strategy was used to isolate genes related to PM-resistance from a PM-resistant clone of Chinese wild *V. quinquangularis*. Approximately 526 clones were sequenced and 492 ESTs were submitted to GenBank. These ESTs were assembled into 266 unigenes and then classified into functional categories of defense, transcription, signal transduction and transport (Table 1). ESTs falling into categories of transcription, signal transduction and transport may be due to a rapid response of these genes following *E. necator*-inoculation or as a result of constitutive expression of these genes in anticipation of pathogen stress. Among them, 205 (77.1%) unigenes appeared to share significant homologies with genes induced by pathogen attack in *V. vinifera*, *A. thaliana*, *N. tabacum* and *O. sativa*, *etc*.

Since SSH was only used to obtain global analysis of gene expression, but not to provide quantitative expression data, real-time qRT-PCR was used to determine the expression levels of some particular genes upon pathogen infection. Expression analysis of 13 selected genes by qRT-PCR revealed that 12 of them were induced more quickly and intensely in the resistant material “Shang-24” than in the susceptible material “Hunan-1” by *E. necator* infection (Figure 4). This transcriptome response in resistant material is consistent with recent findings in the wheat-*Puccinia striiformis* interaction [11] and also with the results that the strong and rapid transcriptional reprogramming involves the induction of defense-related genes in a resistant grapevine (*V. riparia*) upon *Plasmopara viticola* infection, many of which are also induced, albeit to a lesser extent, in a susceptible grapevine (*V. vinifera*) [13]. Polesani *et al.* [13] also reported that the most prevalent functional categories among the modulated transcripts were general metabolism and signal transduction in the resistant grapevine (*V. riparia*). This was very different to our result that the largest category consisting of genes which was predicted to encode resistance and stress-related proteins (Table 1). A possible explanation is that disease resistance involves not only the activation of resistance genes, but also the timing of expression of resistance genes and the associated signal transduction pathways. They only analyzed the early transcriptional changes associated with *P. viticola* infection at 12 and 24 hpi, while we constructed the library with all-stages infection samples of “Shang-24” at the 6, 12, 24, 48, 72, and 120 hpi time points.

It is noteworthy that our finding was quite different from that observed in the grapevine-PM interaction in previous studies [1,4] and from the result in cauliflower that the expression induction of the defense-related genes, such as plant defensin gene *PDF1.2*, lipid transfer protein, and thioredoxin, was quicker and more intense in the susceptible line in response to pathogen, while in resistant line the reaction was delayed and limited [14]. A possible explanation is that resistance in *V. quinquangularis* involves the specific modulation of numerous transcripts that interacted with PM, such as transcripts encoding components of signal transduction cascades, hypersensitive reaction markers and genes involved in jasmonate biosynthesis (Figure 4B), while the limited transcriptional modulation in *V. pseudoreticulata* represents a weak attempted defense response rather than the activation of compatibility-specific pathways observed in *V. vinifera* and the susceptible cauliflower [1,4,14].

### 3.3. Defense-Related Genes

A large number of differentially-expressed cDNAs isolated from the SSH library were classified into several categories and they were involved in a number of physiological and molecular processes, including defense, transcription, signal transduction and transport (Table 1). The largest gene functional category, defense (8.3%), detected in this study was similar to the previous study which categorized disease/defense, accounting for 24% in grapevine infected by *P. viticola* [8]. Moreover, defense-related genes were observed to overlap between this study and the previous study [8] in the susceptible grapevine cultivar Chasselas in response to *P. viticola*, including pathogenesis-related protein, chitinase, lipid transfer protein (LTP), and thaumatin-like protein (TLP) (Table 1). This indicated that there might be common genes in grapevines in response to fungal and oomycete diseases, and putative grapevine defense genes induced in susceptible plants were also induced in resistant plants.

Representatives of this category are genes encoding PR proteins, such as chitinase, TLP, PR-10, and LTP. PR proteins were proven to be associated with plant resistance to various pathogens and abiotic stresses and classified into 17 families [15,16]. In this study, a chitinase gene (UN174) was identified in the SSH library. Chitinases, which belong to the PR-3 family and are an indicator of the systemic acquired resistance (SAR) response, can provide defense against fungal pathogens by degrading fungal chitin following initial penetration of the pathogen into the intercellular space. It has been reported that released chitin might trigger a more generalized defense response in plant tissues [17], therefore the consistently higher expression of the chitinase gene in both resistant and susceptible grapevines in our study suggested that this gene might be involved in generalized defense responses (Figure 4A).

UN073, a class 5 PR protein (PR-5), namely thaumatin-like protein (TLP), was strongly induced (about 60-fold) in resistant grapevine “Shang-24” after *E. necator* inoculation (Figure 4A). PR-5 proteins have been proven to create trans-membrane pores and exhibit antifungal activities *in vitro* by blocking spore germination and germ tube growth of *E. necator*, *Phomopsis viticola* and *Botrytis cinerea* [18]. Moreover, the transgenic expression of TLPs enhanced resistance to several fungal pathogens by inhibiting mycelial growth [19–21].

UN033 presented homology to pathogenesis-related protein 10, which has been identified in many plants. A strong induction of PR-10 was observed in many plants, such as in susceptible grapevine cv. Riesling with *P. viticola* [22], in compatible grapevine cv. Chasselas with *P. viticola* [8] and in rice with *Magnaporthe grisea* [23]. Taking these results together, the induction of PR-10 may play an important role in plant defense against pathogens.

Another type of homologs involved in defense was also identified in this study, namely lipid transfer proteins (UN096, UN170, UN205, and UN226). LTPs are members of class 14 PR protein (PR-14) that are involved in plant defense responses by facilitating lipid transportation to cell walls for the formation of the cuticle and biosynthesis of surface wax. Plant LTPs increase cutin layer thickness to protect leaf cells from fungus damage, retard growth of fungal pathogens and lead to cell death [14,24]. Legay *et al.* [8] found an interesting feature that the LTP structure showed similarity with elicitin: a small lipid-binding protein that is secreted by the phytopathogenic oomycetes *Phytophthora* and *Pythium*, which triggers the hypersensitive response (HR) and SAR in tobacco.

### 3.4. Transport-Related Genes

Twelve transport-related genes were identified in the present study (Table 1), including ABC transporters, responsible for a large number of transport processes. In plants, it has been revealed that toxins, drugs, glutathione conjugates, peptides and secondary metabolites are transported across the membrane via ABC transporters [25]. It was reported that the expression of an *Arabidopsis* ABC transporter (*AtPDR8*) gene was induced by pathogen infection and the gene was a key factor controlling the extent of cell death in the defense response, suggesting that ABC transporters transport some substances, which are closely related to plant response to pathogens [26]. In this SSH library, three genes encoding ABC transporters were identified (Table 1); in light of recent evidence, these genes may be involved in transportation of some substances produced by grapevine or the PM fungus during the infection process.

### 3.5. Ubiquitin/26S Proteasome Pathway

In addition to the direct genes involved in defense, transport, and signal transduction, there were also some other types of genes that potentially relate to the defense response (Table 1), most of which encoded proteins involved in the ubiquitin/proteasome pathway. It appears that ubiquitin-dependent breakdown is emerging as a common regulatory mechanism that not only controls a range of cellular processes in plants, but also plays an important role in plant disease resistance [27]. Recently, JAZ proteins were identified as negative regulators of transcription factors that control the expression of JA-responsive genes. Upon perception of bioactive JA derivatives by the F-box protein, coronatine insensitive 1 (COI1), JAZ proteins were degraded via the conSCF/ubiquitin/26S proteasome pathway, thereby relieving the restrain on JA-responsive genes [28]. COI1, a Leu-rich repeat/F-box protein, determines the substrate specificity of the SCF-type E_3_ ubiquitin-ligase SCF^COI1^, while F-box proteins imparted specificity to the SCF complex through recognizing a specific substrate (typically a regulatory protein), which was then ubiquitinated and subsequently degraded by the 26S proteasome [28]. It is reported that JAZ transcripts increase in response to mechanical wounding and JAZ genes are negatively regulated by one or more labile proteins whose turnover is dependent on COI1 activity [29]. Moreover, it was found that transgenic expression of JAZ1Δ3A or other C-terminally truncated JAZs may provide a useful approach to elucidate specific COI1-dependent processes that confer plant protection to insect herbivores and other forms of environmental stress [29]. In the current study, three genes coding for COI1, F-box family protein, and JAZ protein, respectively, were differentially regulated in the resistant grapevine upon infection by *E. necator*, compared with the susceptible grapevine (Figure 4B), suggesting that these genes may interact with other genes through the ubiquitin/proteasome pathway and contribute to the defense response to *E. necator*.

### 3.6. Photosynthesis and Energy-Related Genes

Approximately 5.6% of the PM-resistance-related genes obtained from the library were identified with a photosynthesis and energy function (Table 1). It is certain that a wide range of physiological and biological changes in a resistant host plant would take place after pathogen inoculation to directly alter growth conditions or represent changes activated in response to pathogen infection [30]. In the current study, genes coding for proteins and enzymes that are related to photosynthetic oxygen evolution (e.g., chlorophyll a/b-binding protein, ribulose-1,5-bisphosphate carboxylase/oxygenase activase, and photosystem components) as reported by Scharte *et al.* [31], have been effectively enriched in the PM responsive library, suggesting that these genes may also play a role in grapevine resistance against *E. necator*.

### 3.7. Metabolism-Associated Genes

It is not surprising that exposure of plants to pathogen infection induces expression of genes encoding proteins involved in metabolic pathways to attenuate the damage of biotic stresses [11]. It has been reported that one of the largest groups of genes activated by pathogen infection comprise genes involved in energy and primary metabolism [11]. In this study, about 21.8% of the identified genes are associated with metabolism, some of which were predicted to encode *S*-adenosylmethionine synthetase (SAMS, UN044), chalcone-flavanone isomerase (CHI, UN182) and phenylalanin ammonia-lyase (PAL, UN260). As a key enzyme of the methionine biosynthesis pathway, SAMS plays an important role in the production of *S*-adenosylmethionine, which is the precursor of ethylene and polyamine biosynthesis pathways. Ethylene has been reported to be involved in a subset of defense responses, and polyamine is considered to pre-induce tolerance of tobacco to different kinds of pathogens [32,33]. In grapevine, accumulation of phytoalexins is the most frequently observed defense reaction upon fungal infection [34]. The grapevine phytoalexins, as flavonoid-type phytoalexins, are formed via the phenylpropanoid pathway, which provides precursors for the biosynthesis of salicylic acid, lignin, and hydroxycinnamic acid amide. Enzymes such as PAL and CHI are crucial to phenylpropanoid metabolism. Several studies indicate that these two enzymes are involved in disease resistance [35,36]. In this study, the expression patterns of three genes encoding SAMS, PAL, and CHI indicate that they might contribute to PM-resistance of grapevine (Figure 4C).

### 3.8. Novel Genes

In our SSH library, 36 (13.5%) unigenes had no hits in the nr protein database; however they showed high homologies to certain DNA sequences in the EST database, most of which were from *V. vinifera*. Four of them were selected for qRT-PCR analysis and the result suggested that three of them showed the similar expression patterns with genes had defense-related roles (Figure 4D). More interestingly, one gene (UN103) was induced more quickly and intensely in the susceptible material “Hunan-1” compared to the resistant material “Shang-24”. The functions of these unigenes in the complex networks of *E. necator*- inoculation processes and whether they contribute to the defense response to *E. necator* in grapevine still await further functional investigation.

In general, more than 200 unigenes have been identified by sequencing the large number of ESTs (Acc. No. JK266423 to JK266914) originating from the grapevine-PM SSH cDNA library obtained in the study, but only 87 more relevant genes were sorted out into different functional categories, based on their putative roles in defense. It is likely that those genes that showed high levels of expression in grapevine “Shang-24” might have relevance, since Chinese wild *Vitis* is a stress-adapted species. Several candidate resistance genes were obtained from resistant material that could be exploited in future biotechnological approaches to increase disease resistance in susceptible grapevine species. The presumed defensive functions of these differentially expressed genes in the complex networks of *E. necator*-resistant processes in grapevine plants await further investigation. Grape resistance to PM is not a single genetic trait, but a complex and systematic network that includes physiological and biochemical processes. By employing qRT-PCR, we have detailed molecular events at the transcriptome level that allowed us to begin unraveling the grapevine defense network in response to *E. necator* infection. Our results suggest that Chinese wild *Vitis* can effectively reprogram its complex signaling networks on a global scale, thus activating expression of multiple genes to mitigate pathogen-induced cellular damage and interfere with pathogen infection processes. Further characterization and functional analysis of these genes will enhance our understanding of the resistance mechanisms of grapevine plants to *E. necator* infection.

## 4. Experimental Section

### 4.1. Plant Material and Inoculation

Chinese wild *V. quinquangularis* clone “Shang-24” (PM-resistant), *V. pseudoreticulata* clone “Hunan-1” (PM-susceptible) and *V. adstricta* Hance clone “Taishan-2” (PM-susceptible) were obtained from the Grape Repository (34°20′N, 108°24′E) of Northwest A & F University, Yangling, Shaanxi, China. The inoculation of *E. necator*, collected from leaves of the field-grown *V. adstricta*, was performed as previously described [4,7]. The inoculated leaves were immediately covered with plastic bags for one night to ensure high humidity. A mock inoculation was made with sterile water at the same time points. Plants were incubated in the greenhouse, with temperatures ranging from 25 to 30 °C and without supplemental lighting. Both treated and mock-inoculated leaves were harvested at 0, 6, 12, 24, 48, 72, 96 and 120 h post inoculation (hpi), respectively, frozen immediately in liquid nitrogen, and stored at −80 °C until use. The experiment was performed with two biological replicates.

### 4.2. Microscopic Observations

*E. necator*-inoculated grapevine leaves of *V. quinquangularis* clone “Shang-24” and *V. pseudoreticulata* clone “Hunan-1” were harvested at 24, 48 and 120 hpi. The *in vivo* detection of H_2_O_2_ during the powdery mildew interaction was carried out using DAB as described in Vanacker *et al.* [12] Three inoculated leaves of each line were fixed at a 24 h interval from 24 to 120 hpi and examined under light microscopy for assessment of epidermal host cell responses. Leaves were fixed and prepared for microscopy by a procedure that avoids displacement of the fungus. For fixation, 3 cm segments cut from the center of inoculated leaves were laid adaxial surface up on filter paper moistened with an ethanol:glacial acetic acid mixture (3:1, *v*/*v*) for 24 to 48 h until the chlorophyll was removed. After bleaching, they were transferred to water soaked filter paper for at least 4 h to relax leaf tissue and finally transferred to papers soaked with lacto-glycerol (1:1:1, lactic acid:glycerol:water, *v*/*v*) for at least 24 h. Segments were stored on lacto-glycerol [12].

For microscopy, the bleached leaf segment was mounted on a microscope slide without a coverslip and observed using a microscope (BX51, Olympus, Tokyo) with a “no coverslip” 100× objective and 20× objective lens. For micrographs, a drop of aniline blue (0.1% [*v*/*v*] in lacto glycerol) was pipetted onto leaf surfaces immediately before they were photographed.

### 4.3. RNA Extraction and SSH Library Construction

For SSH library construction, total RNA of *E. necator*- and mock-inoculated leaves was extracted using the protocol described by Zhang *et al.* [37] with minor modifications in DNase digestion and purification. One microgram of RNA was run on a 1% agarose gel to check the quality. Equimolar amounts of total RNA samples from two corresponding biological replicates and from each time point were combined. The mRNA was isolated from the total RNA, using mRNA purification Kit (Clontech, USA). Two micrograms of high quality mRNA were used for the construction of the library. The purified mRNAs isolated from mock-inoculated grapevine leaves were mixed as the driver, whereas the mRNAs isolated from *E. necator*-inoculated leaves were used as the tester. The SSH cDNA library was constructed using PCR-select cDNA Subtraction Kit (Clontech) following the manufacturer’s instructions. Differentially expressed cDNA fragments digested with *Rsa*I after a two-round PCR selection were cloned into the pGEM-T easy Vector (Promega, WI, USA) and transformed into *E. coli* DH5α (Invitrogen, CA, USA). The clones were cultured overnight on an LB media plate containing ampicillin and X-Gal/IPTG at 37 °C.

### 4.4. Amplification of cDNA Inserts

cDNA inserts from white colonies were PCR-amplified using SP6 and T7 primers. The PCR reactions contained a total volume of 25 μL, including 16 μL sterile water, 2.5 μL 10× buffer, 1.0 μL of each primer (10 μM each), 0.5 μL dNTP mix (2.5 μM each), 0.5 units of *Taq* DNA polymerase (TaKaRa Biotechnology, Dalian, China) and 1 μL of bacterial culture. The PCR was performed as follows: an initial denaturation at 94 °C for 3 min, followed by 35 cycles of 94 °C for 30 s, 60 °C for 30 s and 72 °C for 1.5 min, and a final extension at 72 °C for 5 min. The PCR products were electrophoresed on 1.0% agarose gels to confirm the amplification. A subset of positive clones whose PCR products were longer than 200 bp were selected for preparation of plasmid DNA using the Plasmid DNA extraction kit from U-gene (Anhui, China).

### 4.5. Sequencing and Data Analysis

The selected positive clones were sequenced with the universal M13 sequencing primer. The raw chromatogram files were base-called with Phred [38]. Vector, adaptor and low-quality bases (a 20-bp window with an average error rate >0.01) were trimmed from the raw EST sequences using LUCY [39]. The resulting ESTs were searched against the GenBank nr database and EST sequences of bacterial origin were identified and excluded. The resulting high quality grape ESTs have been deposited in GenBank dbEST database under accession numbers JK266423 to JK266914.

The ESTs were assembled into unigenes using iAssembler [40] with minimum overlap of 40 bp and minimum percent identity of 97. The resulting grape unigene sequences were compared against GenBank non-redundant (nr) and UniProt [41] protein databases using the NCBI BLAST program with a cutoff e value of 1e-5. The unigene sequences were also compared to pfam domain database [42] using HMMER3 [43]. Gene Ontology (GO) terms and plant-specific GO slim ontology [44] were assigned to each unigene, based on terms annotated to its corresponding homologs in the UniProt database and domains in pfam database.

### 4.6. Real Time qRT-PCR Analysis

For qRT-PCR, total RNA extracted from leaves of *E. necator*-inoculated “Shang-24” and “Hunan-1” plants at 0, 6, 12, 24, 48, 72, 96 and 120 hpi as well as from their corresponding mock-inoculated grapevines, was treated with DNase I to remove DNA contamination before cDNA synthesis. The cDNA was synthesized from 1.0 μg total RNA using 500 ng of random hexamers and the M-MLV reverse transcriptase (Promega). cDNAs, derived from inoculated healthy *V. quinquangularis* clone “Shang-24” leaves harvested at 0, 6, 12, 24, 48, 72, 96 and 120 hpi, were used as templates for qRT-PCR. Gene-specific primers for the 13 selected defense-related genes were designed using the Primer Premier 5.0 software. All the primers are listed in Table S2. First-strand cDNA was synthesized from 1 νg of DNase-treated total RNA using PrimeScript™ RTase (TaKaRa Biotechnology).

qRT-PCR reactions were performed in triplicates using iCycler Detection system (BioRad Laboratories, Ltd). Grape Actin1 (GenBank accession no. AY680701) was used as the internal control and for data normalization. A 168 bp fragment of Actin1 gene was amplified with a pair of primers (F: 5′-GAT TCT GGT GAT GGT GTG AGT-3′, R: 5′-GAC AAT TTC CCG TTC AGC AGT-3′). Reactions were performed in a total volume of 20 μL containing 10.0 μL SYBR^®^ Premix Ex *Taq*™ II (TaKaRa Biotechnology), 1.0 μL of cDNA template (6× diluted cDNA from leaf samples), 0.8 μL of each primer (10 pmol·L^−1^), and 7.4 μL ddH_2_O with the following cycling parameters: 95 °C for 30 s, 40 cycles of 95 °C for 5 s, and 60 °C for 30 s. Dissociation curves were generated to ascertain only a single product that was produced in each case. Relative gene expression was determined from standard curves produced using serial cDNA dilutions.

## 5. Conclusions

The results obtained from the present study are the first to provide a global view of gene expression profiles in response to PM pathogen attack in a PM-resistant Chinese wild *V. quinquangularis* accession “Shang-24”. The EST data reported herein provides new insights into the grapevine-PM interaction and the candidate defense-related genes involved in the process. Analyzing gene expression patterns in response to pathogen infection provides a basis for their functional studies.

## Supplementary Materials



## Figures and Tables

**Table 1:** PM-resistance related genes isolated from the SSH library in *V. quinquangularis*.

Unigene No.	Length (bp)	Putative Function	Organism of Best Homology	Accession No.	*E*-Value
**Metabolism (19)**					
UN018	706	Abscisic stress ripening protein	*V. pseudoreticulata*	ABC86744	5 × 10^−21^
UN022	548	GDP-d-glucose phosphorylase	*A. thaliana*	NP_200323	2 × 10^−19^
UN044 a	562	*S*-adenosylmethionine synthetase	*R. communis*	XP_002512570	4 × 10^−101^
UN102	369	ATP-dependent peptidase/ATPase/metallopeptidase	*A. thaliana*	NP_564563	3 × 10^−59^
UN127	829	Malate dehydrogenase	*N. tabacum*	CAC12826	3 × 10^−139^
UN145	540	Putative cinnamoyl-CoA reductase	*R. communis*	XP_002533639	4 × 10^−19^
UN173	250	ATP-dependent peptidase/ATPase/metallopeptidase	*A. thaliana*	NP_568604	9 × 10^−6^
UN178	592	GDSL-motif lipase/hydrolase family protein	*A. thaliana*	NP_190416	7 × 10^−59^
UN183	452	Malate dehydrogenase	*V. vinifera*	AAF69802	6 × 10^−75^
UN230	247	Aldo/keto reductase, putative	*R. communis*	XP_002535506	8 × 10^−18^
UN240	416	*S*-adenosylmethionine-dependent methyltransferase	*A. thaliana*	NP_173458	2 × 10^−63^
UN262	443	HMGB3 (High mobility group B 3)	*A. thaliana*	NP_001031075	3 × 10^−27^
UN266	558	NADH dehydrogenase subunit 2	*A. thaliana*	NP_085584	5 × 10^−16^
UN035	395	Naringenin-chalcone synthase	*Humulus lupulus*	CAK19317	2 × 10^−45^
UN040	645	Cinnamate-4-hydroxylase	*Canarium album*	ACR10242	2 × 10^−99^
UN118	204	Flavonol synthase	*V. vinifera*	BAE75808	1 × 10^−26^
UN166	333	Phenylalanine ammonia-lyase	*Camellia oleifera*	ACT21093	8 × 10^−50^
UN182 a	616	Chalcone-flavanone isomerase	*A. thaliana*	NP_568154	8 × 10^−41^
UN260 a	307	Phenylalanin ammonia-lyase	*V. vinifera*	ABM67591	3 × 10^−25^
**Photosynthesis and Energy (15)**					
UN001	618	Ribulose-1,5-bisphosphate carboxylase/oxygenase activase 1	*Acer rubrum*	ABI94077	2 × 10^−108^
UN017	562	Ribulose bisphosphate carboxylase/oxygenase activase 1	*R. communis*	XP_002532996	2 × 10^−89^
UN020	455	Photosystem I reaction center subunit II, chloroplast precursor	*R. communis*	XP_002516772	4 × 10^−56^
UN023	624	Photosystem II 22 kDa protein, chloroplast precursor	*R. communis*	XP_002513761	1 × 10^−68^
UN025	438	Ribulose bisphosphate carboxylase/oxygenase activase 1	*R. communis*	XP_002532996	3 × 10^−73^
UN052	365	Chloroplast photosystem II 10 kDa polypeptide	*Jatropha curcas*	ADB93062	4 × 10^−41^
UN086	255	Photosystem I reaction center subunit II, chloroplast precursor	*R. communis*	XP_002516772	3 × 10^−17^
UN093	298	Putative fructose-1,6-bisphosphatase	*R. communis*	XP_002527886	3 × 10^−28^
UN095	348	Ribulose-1,5-bisphophate carboxylase/oxygenase activase 1	*V. pseudoreticulata*	ABC86738	5 × 10^−47^
UN098	571	Putative fructose-bisphosphate aldolase	*R. communis*	XP_002512993	2 × 10^−100^
UN106	268	Ribulose-1,5-bisphosphate carboxylase/oxygenase activase 1	*Gossypium hirsutum*	ABB20913	2 × 10^−20^
UN121	371	Chlorophyll A/B binding protein	*R. communis*	XP_002533251	1 × 10^−45^
UN198	312	Light harvesting chlorophyll a/b-binding protein	*Nicotiana sylvestris*	BAA25394	2 × 10^−35^
UN241	189	Light-harvesting complex II protein Lhcb1	*Populus trichocarpa*	XP_002307725	4 × 10^−11^
UN255	324	Chlorophyll A/B binding protein	*R. communis*	XP_002519950	5 × 10^−44^
**Disease/Defence (22)**					
UN016	477	Stress-induced cysteine proteinase	*Lavatera thuringiaca*	AAB62937	4 × 10^−66^
UN033	427	Similar to pathogenesis-related protein 10.3	*V. vinifera*	XP_002274617	1 × 10^−51^
UN040	645	Cytochrome P450	*Populus trichocarpa*	XP_002325637	3 × 10^−98^
UN073 a	351	Thaumatin-like protein	*V. vinifera*	AAQ10092	7 × 10^−67^
UN075	346	Glutathione *S*-transferase 3	*Papaver somniferum*	AAF22519	1 × 10^−8^
UN077	450	Chaperone protein dnaJ	*R. communis*	XP_002514419	3 × 10^−68^
UN079	294	Responsive to dehydration 19	*A. thaliana*	NP_568052	2 × 10^−43^
UN096	755	Lipid transfer protein isoform 4	*V. vinifera*	AAO33394	3 × 10^−47^
UN170	468	Lipid transfer protein family protein	*Tamarix hispida*	ACM78616	6 × 10^−8^
UN205	470	Lipid transfer protein family protein	*A. thaliana*	NP_680546	5 × 10^−31^
UN226	537	Lipid transfer protein family protein	*A. thaliana*	NP_565348	2 × 10^−33^
UN115	574	Bacterial-induced peroxidase precursor	*G. hirsutum*	AAD43561	1 × 10^−71^
UN116 a	769	Similar to senescence-associated protein	*A. thaliana*	BAD42919	3 × 10^−70^
UN119	698	Catalase 1	*N. tabacum*	AAB71764	2 × 10^−80^
UN174 a	443	Class IV chitinase	*N. tabacum*	BAF44533	3 × 10^−41^
UN179	318	Proline-rich protein	*Solanum tuberosum*	CAA04449	1 × 10^−22^
UN209	212	Harpin-induced family protein	*A. thaliana*	NP_190008	1 × 10^−22^
UN219	341	Responsive to dehydration 21	*A. thaliana*	NP_564497	2 × 10^−44^
UN233	465	Proline-rich protein	*G. hirsutum*	ABM05952	2 × 10^−16^
UN239	240	Chitinase-like protein	*G. hirsutum*	AAQ56598	2 × 10^−35^
UN058	474	2OG-Fe(II) oxygenase family protein	*A. thaliana*	NP_192787	1 × 10^−50^
UN043	581	Ascorbate peroxidase 3	*A. thaliana*	NP_195226	4 × 10^−67^
**Protein synthesis and fate (7)**					
UN004	372	40S ribosomal protein S17	*A. thaliana*	NP_196100	1 × 10^−41^
UN010	252	26S proteasome regulatory subunit	*A. thaliana*	AAP86657	2 × 10^−39^
UN134 a	468	Coronatine insensitive 1 (COI1)	*A. thaliana*	ABR45948	2 × 10^−47^
UN135 a	356	F-box family protein	*A. thaliana*	NP_199429	2 × 10^−31^
UN203 a	784	Jasmonate ZIM-domain protein 2	*N. tabacum*	BAG68656	5 × 10^−43^
UN214	352	Putative 40S Ribosomal protein	*O. sativa*	AAK92638	3 × 10^−39^
UN254	229	Aspartyl protease family protein	*A. thaliana*	NP_566966	3 × 10^−15^
**Signal transduction (8)**					
UN005	653	Putative zinc finger protein	*O. sativa*	BAD87736	1 × 10^−24^
UN008	415	CIPK2; protein serine/threonine kinase	*A. thaliana*	NP_196324	2 × 10^−11^
UN031	410	Serine/threonine phosphatase	*A. thaliana*	NP_188632	5 × 10^−29^
UN151	332	CIPK21; protein serine/threonine kinase	*A. thaliana*	NP_568860	3 × 10^−29^
UN211	636	Lipid phosphate phosphatase 3	*A. thaliana*	NP_001078096	2 × 10^−35^
UN216	447	GTP binding/phospholipase activator	*A. thaliana*	NP_191788	1 × 10^−79^
UN217	508	Rab-type small GTP-binding protein	*Cicer arietinum*	BAA76423	2 × 10^−49^
UN238	543	Phosphoribulose kinase, putative	*R. communis*	XP_002519002	3 × 10^−82^
**Transport (12)**					
UN011	324	NRAMP3; transmembrane transporter	*A. thaliana*	NP_179896	6 × 10^−45^
UN029	862	Plasma membrane aquaporin	*V. vinifera*	AAF80557	2 × 10^−148^
UN030	910	Antiporter/triose-phosphate transmembrane transporter	*A. thaliana*	NP_851138	1 × 10^−144^
UN042	584	Putative sorbitol transporter	*Prunus cerasus*	AAM44082	7 × 10^−91^
UN067	647	Non-specific lipid transfer protein	*V. vinifera*	ABA29446	2 × 10^−36^
UN088	249	Plasma membrane intrinsic protein	*A. thaliana*	AAB65787	3 × 10^−38^
UN129	401	ABC transporter family protein	*Populus trichocarpa*	XP_002331473	6 × 10^−60^
UN136	478	ABC transporter-like	*A. thaliana*	BAF01914	3 × 10^−83^
UN231	565	ABC transporter family protein	*Populus trichocarpa*	XP_002308160	7 × 10^−56^
UN164	509	Polyol transporter	*Populus trichocarpa*	XP_002313809	4 × 10^−68^
UN223	269	Nitrate transporter	*Malus hupehensis*	ACN72639	2 × 10^−34^
UN221	329	Solute symporter family protein	*A. thaliana*	NP_199351	5 × 10^−42^
**Transcription (4)**					
UN014	517	Transcription activator/transcription factor	*A. thaliana*	NP_197904	2 × 10^−18^
UN064	446	Transcript elongation factor	*A. thaliana*	NP_181390	5 × 10^−27^
UN065	443	IAA16; transcription factor	*A. thaliana*	NP_187124	3 × 10^−46^
UN167	430	DNA binding/transcription factor	*A. thaliana*	NP_172094	1 × 10^−21^

a Clones selected for further qRT-PCR analysis are marked with an asterisk.

## Abbreviations

ABC: ATP-binding cassette
CHI: chalcone-flavanone isomerase family protein
COI1: coronatine insensitive 1
EST: expressed sequence tag
GO: gene ontology
hpi: hour post inoculation
JAZ: jasmonate ZIM-domain protein
LTP: lipid transfer protein
PAL: phenylalanine ammonia-lyase
PM: powdery mildew
qRT-PCR: quantitative reverse transcriptase polymerase chain reaction
SAMS: *S*-adenosylmethionine synthetase
SAR: systemic acquired resistance
SSH: suppression subtractive hybridization
