# Supplementary material for: Characterization of Erysiphe necator-Responsive Genes in Chinese Wild Vitis quinquangularis
Source: Int J Mol Sci. 2012 Sep 12;13(9):11497–519. doi: 10.3390/ijms130911497 (PMC3472759; doi:10.3390/ijms130911497)
Supplement: Supplementary file 1 [file ijms-13-11497-s002.pdf]

Supplementary Information

Table S2. Primer sequences used for gene expression analysis by qRT-PCR.

Unigene No.	Gene Name		Primers Sequence (5'–3')	Product Length (bp)
UN044	SAMS	F	GAGACTTGCACGAAGACCAACA	125
		R	CCCACATCGTCAGATACAAATCC	
UN073	Thaumatin-like protein	F	TCGCACTTAACCAATTCAGCA	90
		R	TGGAAGTAGGATTAAAGGCCGTAG	
UN116	SAP	F	CGGCTACATCCACCAACTCT	122
		R	AACCTTCTTCCAATCACGCTTC	
UN134	COI1	F	AGATGAGGGGCTGTTGCTTC	92
		R	TCTATACCCTTGCACCCACAAA	
UN135	F-box family protein	F	GTGTCTGATGGATCTTGGGTTTC	133
		R	CCCTCATTCTTCTCCTCCACTATTC	
UN174	Class IV chitinase	F	AAATGCTTCCCTTCGCAGTT	85
		R	GTGTCTCCGTTGCTGATGTTG	
UN182	CHI	F	TCCAGGTCAAGTTCACAGCAA	87
		R	CCTTTCCAGATTTACCCTTCCA’	
UN203	JAZ2	F	GGCACATTCAGGGGATTTCTT	90
		R	GCATCCTTGGCTCGGTTTT	
UN260	PAL	F	CAATCCAGAAGAGGGTCGATG	102
		R	AGGGCTGCTGACTGGTGAA	
UN113	no hits found	F	GGGCATTCAGTGCTGGATTTA	158
		R	GCGGCTTTGGTCTTTGATTTG	
UN168	no hits found	F	GGAAGGGTTCGTGGGAGGTT	269
		R	CCAAAATGAACTGGAAGCAGAAGA	
UN235	no hits found	F	CATCTCCTCAGCCCACAATTAGC	193
		R	TGATCGTCAGTCGCAAGAAAGC	
UN103	no hits found	F	GCAAGAGCTGTGGAATTTATCGT	147
		R	TTGTTGTTGGTCCGTAGTGGC	
